# Triple negative breast cancers comprise a highly tumorigenic cell subpopulation detectable by its high responsiveness to a Sox2 regulatory region 2 (SRR2) reporter

**DOI:** 10.18632/oncotarget.3590

**Published:** 2015-03-14

**Authors:** Karen Jung, Nidhi Gupta, Peng Wang, Jamie T. Lewis, Keshav Gopal, Fang Wu, Xiaoxia Ye, Abdulraheem Alshareef, Bassam S. Abdulkarim, Donna N. Douglas, Norman M. Kneteman, Raymond Lai

**Affiliations:** ^1^ Department of Oncology, University of Alberta, Edmonton, Alberta, Canada; ^2^ Department of Laboratory Medicine and Pathology, University of Alberta, Edmonton, Alberta, Canada; ^3^ Department of Surgery, University of Alberta, Edmonton, Alberta, Canada; ^4^ Department of Oncology, McGill University, Montreal, Quebec, Canada; ^5^ DynaLIFE_Dx_ Medical Laboratories, Edmonton, Alberta, Canada

**Keywords:** breast cancer, tumour cell heterogeneity, Sox2, SRR2

## Abstract

We have recently described a novel phenotypic dichotomy within estrogen receptor-positive breast cancer cells; the cell subset responsive to a Sox2 regulatory region (SRR2) reporter (RR cells) are significantly more tumorigenic than the reporter unresponsive (RU) cells. Here, we report that a similar phenomenon also exists in triple negative breast cancer (TNBC), with RR cells more tumorigenic than RU cells. First, examination of all 3 TNBC cell lines stably infected with the SRR2 reporter revealed the presence of a cell subset exhibiting reporter activity. Second, RU and RR cells purified by flow cytometry showed that RR cells expressed higher levels of CD44, generated more spheres in a limiting dilution mammosphere formation assay, and formed larger and more complex structures in Matrigel. Third, within the CD44^High^/CD24^−^ tumor-initiating cell population derived from MDA-MB-231, RR cells were significantly more tumorigenic than RU cells in an *in vivo* SCID/Beige xenograft mouse model. Examination of 4 TNBC tumors from patients also revealed the presence of a RR cell subset, ranging from 1.1-3.8%. To conclude, we described a novel phenotypic heterogeneity within TNBC, and the SRR2 reporter responsiveness is a useful marker for identifying a highly tumorigenic cell subset within the CD44^High^/CD24^−^tumor-initiating cell population.

## INTRODUCTION

Triple negative breast cancer (TNBC), accounting for 10 to 20% of all breast tumors, is characterized by the absence of estrogen receptor, progesterone receptor, and Her2. The subtype lack effective targeted therapies, and exhibit poor prognosis.

Sox2 is a transcription factor important in maintaining the pluripotency of embryonic stem cells [1]. Its expression is largely restricted to embryonic stem cells and somatic stem cells [1], including the breast stem/progenitor cells [2, 3]. In breast cancer, aberrant expression of Sox2 is detected in up to 30% of the tumors detectable by immunohistochemistry [4], and this aberrancy correlates with larger tumor size, higher tumor grade [5], and lymph node metastasis [6]. Experimentally, it was demonstrated that enforced expression of Sox2 in breast cancer cells contributes to enhanced proliferation and invasion *in vitro*, and tumor formation in xenograft mouse models [4, 5]. In studies reported by us, we found that the transcriptional activity of Sox2, detectable by the Sox2 regulatory factor-2 (SRR2) reporter, is found only in a small subset of cells in estrogen receptor-positive breast cancer cell lines and patient samples [7, 8]. This has since been confirmed in studies by other groups [9, 10]. Importantly, we also found that cells showing reporter responsiveness (i.e. RR cells) display significantly higher tumorigenic capacity than those that are reporter unresponsive (i.e. RU cells) [7].

Here, we report that the dichotomy of RU and RR cells also exists in TNBC. Importantly RR cells are significantly more tumorigenic than their RU counterparts *in vitro* and *in vivo*, which is evident in the CD44^High^/CD24^−^tumor-initiating cell population.

## RESULTS

### TNBC cell lines comprise cells with heterogeneous SRR2 reporter activity

**Figure 1:**
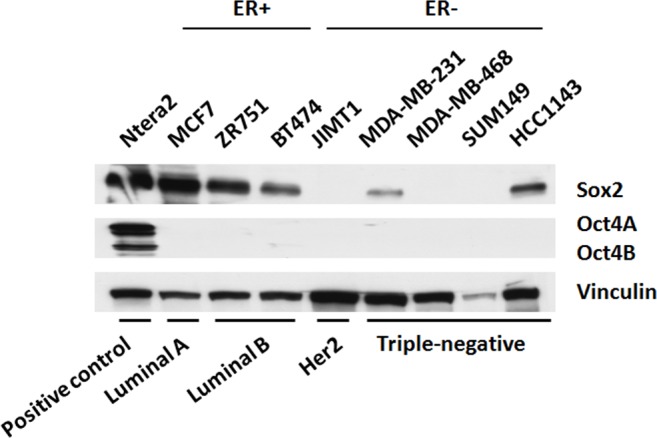
Sox2 expression is low or undetectable in triple negative breast cancer cell lines Western blot depicting Sox2, and Oct4A/B protein expression across ER− and ER+ breast cancer cell lines. Ntera2, a malignant human pluripotent embryonic carcinoma cell line, acts as a positive control for Sox2 and Oct4A/B.

As shown in the upper panel of Figure 1, western blot studies of eight breast cancer cell lines showed that Sox2 is expressed in 3 of 3 ER+ cell lines (MCF7, ZR751 and BT474) as well as 2 of 4 TNBC cell lines (MDA-MB-231 and HCC1143). JIMT (Her2-positive) and 2 of 4 TNBC cell lines (MDA-MB-468 and SUM149) showed no detectable Sox2. The Sox2 expression levels in the two Sox2-positive TNBC cell lines were generally lower than those of the estrogen receptor-positive cell lines. We asked if Oct4, a Sox2 co-factor in ESCs [1], is also expressed in these breast cancer cell lines. As shown in the middle panel of Figure 1, no detectable Oct4A or Oct4B was found in all cell lines examined. Ntera, a human teratocarcinoma cell line, served as the positive control for both Sox2 and Oct4 detection.

**Figure 2:**
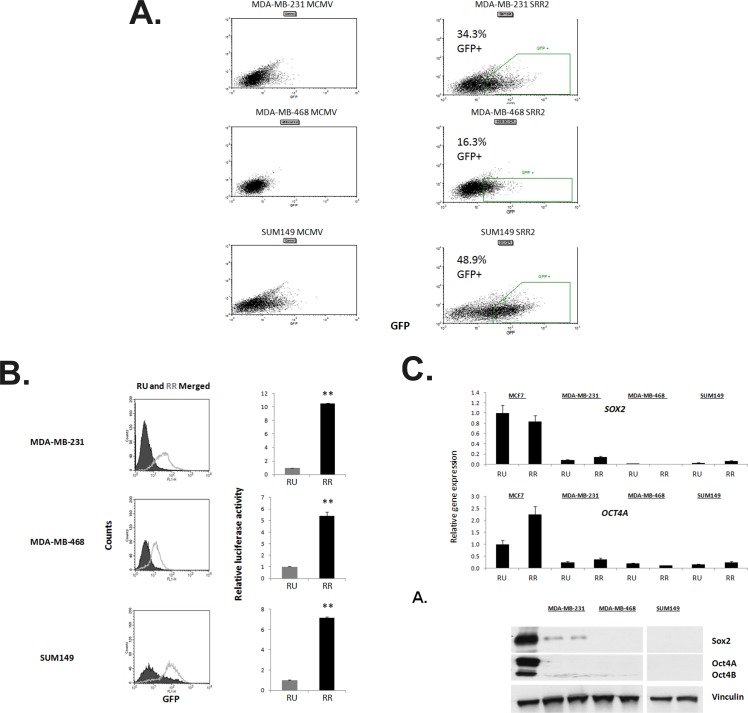
TNBC cell lines comprise of cells with heterogeneous Sox2 regulatory region 2 (SRR2) reporter activity A. FACS dot plots illustrating the GFP expression of ER− cell lines virally-infected with the mCMV or SRR2 reporter plasmids. Gates drawn show the RU and RR subsets collected and cultured separately thereafter, percent of gated live population is reported. B. Flow cytometry dot plot and merged histogram analyses for GFP expression of ER− RU and RR lines. Cells were also harvested and assayed for relative SRR2 luciferase activity. C. Q-PCR results of *SOX2* and *OCT4A* expression in the triple-negative RU and RR cell lines normalized to *GAPDH*, and further normalized to MCF7 RU sample. Previously reported high Sox2-expressing MCF7 RU and RR cell lines *SOX2* and *OCT4A* expression data are shown for comparison. Western blot visualizing Sox2 and Oct4A/B protein expression. Ntera2 (a malignant human pluripotent embryonic carcinoma cell line) acts a positive control for Sox2 and Oct4A/B expression.

To facilitate our studies, we established TNBC cell clones stably transfected with the SRR2 reporter using a lentiviral infection protocol described previously [7]. Three TNBC cell lines, including MDA-MB-231, MDA-MB-468 and SUM-149, were included for this study. Cells from these three cell lines stably transfected with the minimal CMV reporter served as the negative controls. To detect evidence of responsiveness to the SRR2 reporter, we performed flow cytometry to detect GFP expression. At two weeks after the lentiviral infection, all three cell lines showed reporter responsiveness in a subset of cells, with 34.3% in MDA-MB-231, 16.3% in MDA-MB-468 and 48.9% in SUM149, as compared to the mCMV reporter cells (Figure 2A).

Using a flow cytometry cell sorter, we purified reporter unresponsive (RU) cells and reporter responsive (RR) cells based on their differential GFP expression, and the gating strategy is illustrated in Supplemental Figure 1. Specifically, to establish the RR cell clones for each of these cell lines, we isolated approximately 5% of cells showing the highest level of GFP. Purified RU and RR cells were cultured and expanded separately. At 8 weeks after the lentiviral infection, we performed flow cytometry and confirmed that RU cells remained GFP-negative and RR cells were highly enriched in GFP-positive cells, with 92.7% in MDA-MB-231, 64.8% in MDA-MB-468, and 83.1% in SUM149 (Figure 2B). Correlating with these findings, RR cells had significantly higher luciferase activity than RU cells, as shown in the right panel of Figure 2B. This phenotype was stable for all experiments, and the cells were not kept beyond 10 passages from lentiviral infection.

To exclude the possibility that the lack of GFP or luciferase expression in RU cells is due to the absence of the SRR2 reporter construct, we amplified the *gfp* gene included in the reporter using PCR. As shown in Supplemental Figure 2, we were able to detect the *gfp* gene in the RU, RR, unsorted cells stably infected with the SRR2 reporter, and cells infected with the minimal CMV (negative control).

### *Sox2* is not a major contributor in driving the SRR2 reporter activity in TNBC cells

By quantitative PCR and western blot, we confirmed that the established RU and RR cells derived from the three TNBC cell lines exhibited very low expression levels of *SOX2*, compared to the estrogen receptor-positive breast cancer cell lines (Figure 2C). This finding was in parallel with that of the parental cell lines (Figure 1). Again, Oct4A was not detectable (Figure 2C). Western blot studies showed similar results (Figure 2C). Sox2 siRNA knockdown in RU and RR cells paradoxically increased luciferase activity (Supplemental Figure 3). Further, enforced robust expression of Sox2 into RU cells did not significantly increase their luciferase activity, while the same treatment increased the luciferase activity in RR cells by only 1.5-folds (Supplemental Figure 3). These observations support the concept that Sox2 is not a major contributor to the SRR2 reporter activity in TNBC cells.

### RR cells exhibit higher CD44 expression, enhanced capacities for colony formation *in vitro*, and higher frequency of mammosphere-forming cells

**Figure 3:**
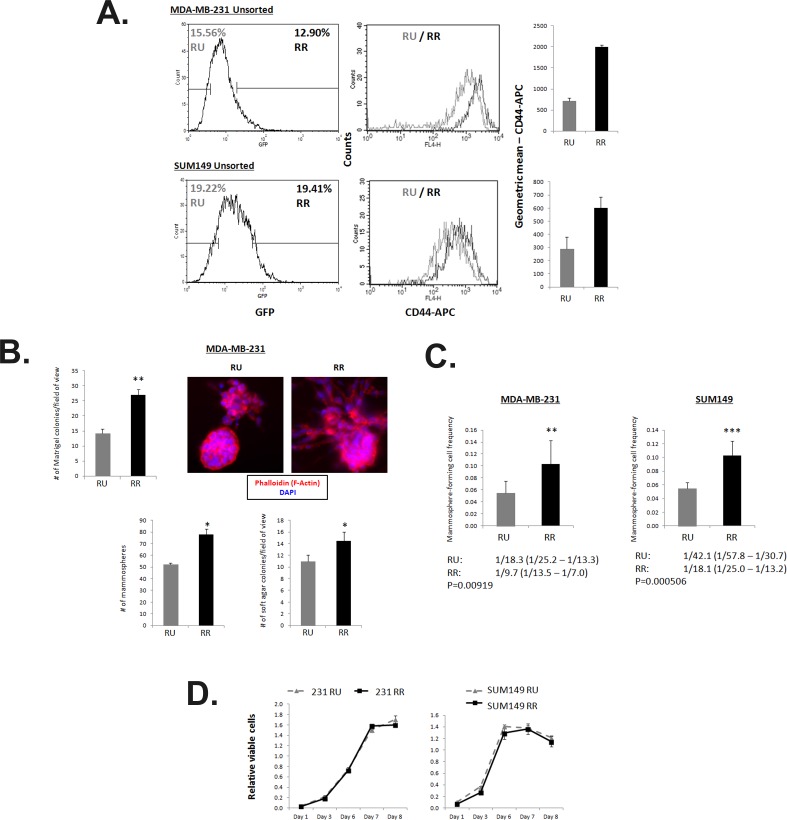
RR cells exhibit higher CD44 expression, enhanced capacities for colony formation *in vitro* and higher frequency of mammosphere-forming cells A. Flow cytometry analyses of MDA-MB-231 and SUM149 Unsorted SRR2 cells stained with CD44-APC. Cells were gated on the highest and lowest 10 to 20% GFP expression and analyzed for CD44-APC levels. B. Results for Matrigel colony formation assay, conventional mammosphere assay, and soft agar assay of untreated MDA-MB-231 RU and RR cells are shown. 2500 cells/well are seeded into a 96-well Matrigel colony formation assay and colonies are counted from photographs taken on Day 7. Photographs of Matrigel multi-cell colonies were stained with phalloidin and imaged by high content screening imaging microscopy. 10,000 cells/well are seeded into a 6-well mammosphere assay and counted on Day 7. 10,000 cells/well are seeded into a 24-well soft agar assay and counted on Day 28. C. Extreme limiting dilution analyses statistics and graphical depiction of results are shown of a limiting dilution mammosphere assay in a 96-well plate format. Cells were seeded in 10 seeding densities ranging from 1 to 1000 cells/well in 6 replicates each. D. MTS 2-dimensional proliferation assay quantification of untreated ER− RU and RR cells seeded at 2000 cells/well. 20 μL of MTS reagent is added with fresh media 2 hours prior to taking absorbance reading.

Using the established purified RU and RR cell clones derived from MDA-MB-231 and SUM149, we assessed the biological significance of the differential responsiveness to the SRR2 reporter. As shown in Figure 3A, CD44 is 2-fold higher in RR cells as compared to RU cells. In a Matrigel colony formation assay, we found that RR cells formed significantly more colonies (1.5X) than RU cells did; furthermore, the colonies formed by RR resulted in more complex multi-cellular structures, with a greater number of multi-cellular extensions protruding from the colonies into the Matrigel (Figure 3B). Compared to RU cells, RR cells also formed significantly more spheres (1.5X) in a mammosphere assay, and significantly more colonies (1.5X) in a soft agar assay (Figure 3B).

To further compare the mammosphere forming ability of the RU and RR cells, we used a 96-well limiting dilution mammosphere formation assay and found that the RR cells derived from MDA-MB-231 exhibited a mammosphere-forming cell frequency of 1/9.7, as compared to 1/18.3 in RU cells (p=0.00919). Similarly, RR cells derived from SUM149 exhibited a mammosphere-forming cell frequency of 1/18.1 cells, as compared to 1/42.1 for RU cells (p=0.000506) (Figure 3C). Of note, these phenotypic differences between RU and RR cells shown in various *in vitro* assays are not due to their differential rates of cell proliferation, as the 2-dimensional proliferation of RU and RR cells were comparable, as shown by the MTS assay (Figure 3D).

### SRR2 reporter activity is a novel marker to enrich for a more tumorigenic cell subset within the CD44^High^/CD24- population

**Figure 4:**
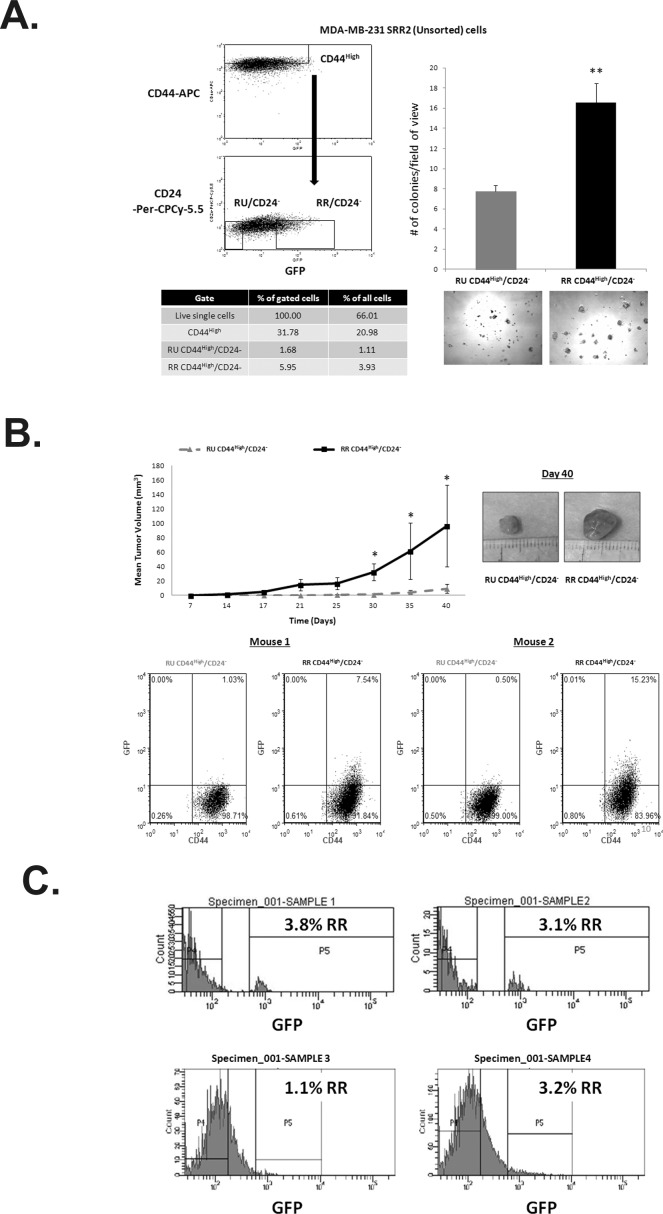
SRR2 reporter activity is a novel marker to enrich for a more tumorigenic cell subset within the CD44/CD24 population A. FACS dot plot showing the sorting scheme of the RU CD44^High^/CD24^Neg^ and RR CD44^High^/CD24^−^ subsets. Percentages of gated populations from the live single cell population are reported. Cells were subsequently collected and seeded at 2500 cells/well in a 96-well Matrigel colony formation assay. Photographs were taken at 5X objective on Day 7. B. Purified RU CD44^High^/CD24^−^ and RR CD44^High^/CD24^−^ cell subsets as described above from MDA-MB-231 SRR2 cells were resuspended in 1:1 Matrigel/PBS. 4000 cells were injected with 200 μL of Matrigel/PBS solution subcutaneously bilaterally into 6-8 week old SCID/Beige females. Photographs depict representative tumors at Day 40. Resultant tumors were dissociated and analyzed by flow cytometry for GFP and CD44 expression. Representative 2 of 6 mice shown. C. Fresh TNBC patient tumors were dissociated, infected with the lentiviral SRR2 reporter, and assessed for GFP by flow cytometry.

Next, we asked if the SRR2 reporter activity is a useful marker to isolate a more robust tumorigenic subset within the CD44^High^/CD24^−^ tumor-initiating cell population [11]. RU and RR derived from MDA-MB-231 were used for these experiments. As shown in Figure 4A, within the CD44^High^/CD24^−^ population, RR cells gave rise to significantly more colonies (2X) in Matrigel (Figure 4A). We then performed SCID/Beige mouse xenograft assay using RU and RR cells within the CD44^High^/CD24^−^ cell population. As shown in Figure 4B, RR cells were significantly more tumorigenic *in vivo*, forming significantly larger tumors within 6 weeks after xenografting. Moreover, upon dissociation of the resultant xenograft tumors, we found that the tumors derived from RR cells comprised mostly GFP-negative cells and a small subset of GFP^low^ cells suggesting that RR gave rise to RU cells *in vivo* (Figure 4B). In comparison, RU cells were homogeneously GFP-negative (Figure 4B).

### SRR2 reporter activity is detectable in TNBC primary patient samples

Finally, we asked if the dichotomy of RU and RR also exists in primary patient samples. Four cases of fresh, previously untreated TNBC patient samples were processed and infected with the SRR2 reporter using a protocol described previously [8]. As shown in Figure 4C, we detected a small (1.1 to 3.8%) RR cell subset in all cases examined. Due to low cell numbers, the patient RR cells were not further characterized.

## DISCUSSION

The key finding of this study is that we have shown that the SRR2 reporter is a useful marker for identifying a novel dichotomy in TNBC, with RR cells being more tumorigenic than RU cells *in vitro*. Importantly, within the CD44^High^/CD24^−^ tumor-initiating cell population derived from MDA-MB-231, RR cells were found to be significantly more tumorigenic than RU cells in an *in vivo* SCID/Beige xenograft mouse model.

The obvious question arising from our observations is how the SRR2 reporter responsiveness is linked to the high tumorigenic potential. Unlike estrogen receptor-positive breast cancer cells, Sox2 is not a major contributor to the reporter responsiveness. While the mechanism underlying the reporter responsiveness in TNBC is under active investigation in our laboratory, our initial bioinformatics analysis of the SRR2 reporter has revealed potential binding sites for multiple transcriptional factors such as C-Myc and Stat3. It is likely that one or more of these transcriptional factors contributes to the SRR2 reporter responsiveness and high tumorigenicity, potentially serving as therapeutic targets for TNBC. Overall, we believe that our experimental model is useful in studying the biology of breast cancer stemness.

While RR cells in tissue culture retained reporter responsiveness, as evidenced by their relatively constant GFP-positivity, xenografts derived from RR cells were composed of mostly RU cells. This finding was consistent among all 6 xenografts examined. We speculate that RR cells gave rise to RU cells *in vivo*. Moreover, RU cells remained to be GFP-negative. This would be in keeping with the concept that the RR cell subset is enriched in cancer stem cells.

## MATERIALS AND METHODS

### Cell lines, reagents, and western blotting

MDA-MB-231, MDA-MB-468, and Ntera2 were purchased from ATCC and cultured in DMEM high glucose media supplemented with 10% FBS. SUM149 cells were obtained from Dr. Sandra E. Dunn (University of British Columbia) through a collaboration and were cultured in F12 media supplemented with 5% FBS, 5 μg/mL insulin, 1 μg/mL hydrocortisone, and 10 mM Hepes. Cell lines were virally infected twice with mCMV or SRR2 reporter as previously described [7]. Successfully infected cells were selected with and maintained in puromycin as previously described [7]. Antibodies used: Sox2 XP (1:500, #3579) from Cell Signaling; Oct4 (1:500, #sc-5279) and Vinculin (1:1000, #sc-7649) from Santa Cruz. Vinculin acts as a loading control for all western blots.

### Fluorescence-activated cell sorting (FACS) and flow cytometry analyses

Single cell suspensions for FACS and flow cytometry are achieved by passing cells through 40 μm cell stainer (BD Falcon) and staining with CD44-APC (#559942) and CD24-PerCP-Cy5.5 (#561647) from BD Pharmingen in Hanks' buffer supplemented with 2% FBS. Cells were collected in Hanks' buffer supplemented with 50% FBS.

### Genomic DNA extraction, PCR, quantitative PCR, and SRR2 reporter luciferase assay

DNA was extracted using the DNeasy Blood and Tissue Kit (Qiagen) and the *gfp* gene was amplified with Taq polymerase (Invitrogen) and primers as previously described. *Gfp* primers: F – AGGACAGCGTGATCTTCACC, R – CTTGAAGTGCATGTGGCTGT. Quantitative PCR and SRR2 luciferase assay were performed as previously described. *SOX2* specific qPCR primer sequences: F – GCTACAGCATGATGCAGGACCA, R – TCTGCGAGCTGGTCATGGAGTT. *OCT4A* specific qPCR primer sequences: F – CTTCTCGCCCCCTCCAGGT, R – AAATAGAACCCCCAGGGTGAGC [12].

### Matrigel assay and MTS proliferation assay

For the Matrigel assay, cells were seeded at 2500 cells/well in 200 μL of media atop of 40 μL of Corning Matrigel matrix in 96-well plate, pictures taken on Day 7. U0126, EGF, or vehicle controls were added directly into media and incubated for the full 7-day assay duration. The MTS assay was measured with 2000 cells seeded. On day of quantification, 100 μL media was added with 20 μL of MTS reagent (Promega) and the optical density read after a 2-hour incubation.

### Matrigel colony F-actin staining and imaging

Matrigel assays were performed as described above and stained using a previously published protocol for fixing and imaging whole Matrigel cultures without extraction [13].

### Limiting dilution and conventional mammosphere formation assay

Cell were trypsinized and passed through a 40 μm cell strainer (BD Falcon) and seeded in Mammocult media and supplements (StemCell Technologies) in 96-well low-adherent plate (Corning) at 10 limiting dilutions ranging from 1 to 1000 cells. Each dilution had 6 replicates each, and each well was scored for presence or absence of spheres after 7 days. Data was analyzed using the Extreme Limiting Dilution Analysis (ELDA) software for 3 independent experiments [14].

### Xenograft tumor formation assay and animal care

Recipient animals (SCID/Beige) were housed virus/antigen free, and cared for in accordance with Canadian Council on Animal Care guidelines. Experimental protocols were reviewed and approved by the University of Alberta Health Sciences Animal Welfare Committee. Freshly FACS-purified RU CD44^High^/CD24^−^ and RR CD44^High^/CD24^−^ cell subsets from MDA-MB-231 SRR2 cells were resuspended in 1:1 Matrigel/PBS. 4000 cells were injected with 200 μL of Matrigel/PBS solution subcutaneously bilaterally into 6-8 week old SCID/Beige females (Taconic). Mice were monitored for tumor size and weight twice weekly. Tumor volume (V) in mm^3^ was calculated using the following formula: V = [length x width^2^]/2. For tumor growth statistics, non-parametric Mann-Whitney test was carried out using SPSS (Version 16) software to compare tumor volume between two groups. P < 0.05 was considered statistically significant. Resultant tumors were dissociated for flow cytometry analyses as previously described [8].

### Primary patient tumor cells analyses

Fresh patient tumors were processed and analyzed as previously described [8].

## SUPPLEMENTARY MATERIAL FIGURES


