# Supplementary material for: Triple negative breast cancers comprise a highly tumorigenic cell subpopulation detectable by its high responsiveness to a Sox2 regulatory region 2 (SRR2) reporter
Source: Oncotarget. 2015 Mar 14;6(12):10366–73. doi: 10.18632/oncotarget.3590 (PMC4496361; doi:10.18632/oncotarget.3590)
Supplement: Supplementary file 1 [file oncotarget-06-10366-s001.pdf]

Triple negative breast cancers comprise a highly tumorigenic cell subpopulation detectable by its high responsiveness to a Sox2 regulatory region 2 (SRR2) reporter

Supplementary Material

SUPPLEMENTARY FIGURE 1

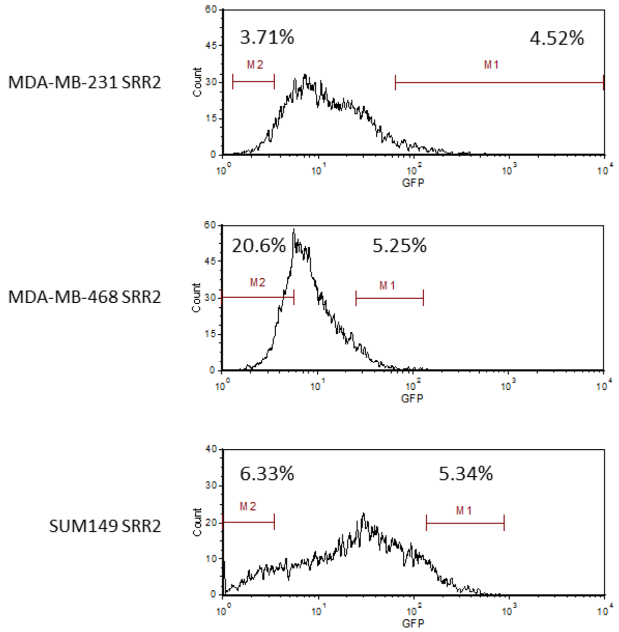

1

Supplemental Figure 1: TNBC RR cells were purified by isolating the cells with the highest 5% GFP expression. FACS dot plots illustrating the GFP expression of ER- cell lines virally-infected with the mCMV or SRR2 reporter plasmids. Gates drawn show the RU and RR subsets collected and cultured separately thereafter, percent of gated live population is reported.

SUPPLEMENTARY FIGURE 2

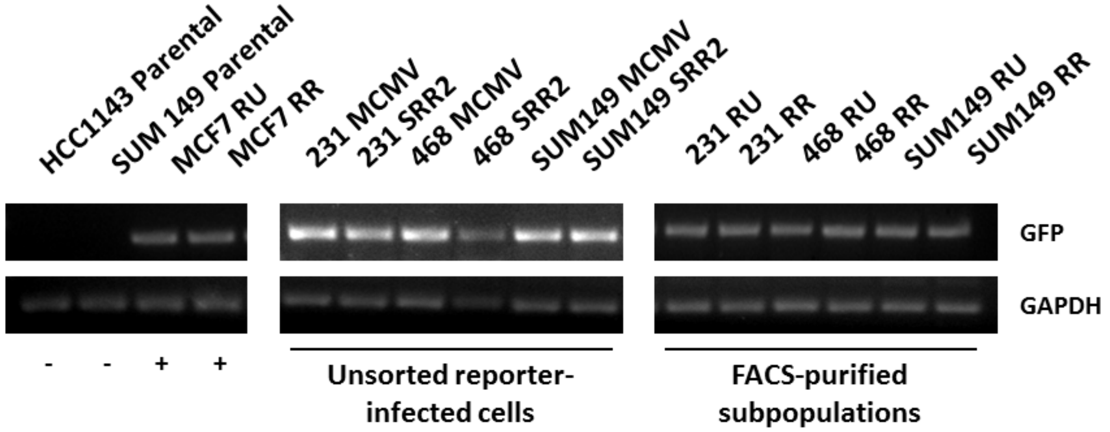

2

Supplemental Figure 2: RU cells did not lose the SRR2 reporter. Agarose gel results of PCR experiments amplifying *gfp* and *GAPDH* genes of genomic DNA extracted from ER- cell lines virally infected with mCMV or SRR2 reporter plasmids and the FACS-purified RU and RR populations. The lines had been passaged and cultured for 8 weeks post-viral infection at the time of genomic DNA extraction. Parental breast cancer cell lines and previously reported SRR2 reporter-expressing MCF7 RU and RR cell lines act as negative and positive controls respectively.

SUPPLEMENTARY FIGURE 3

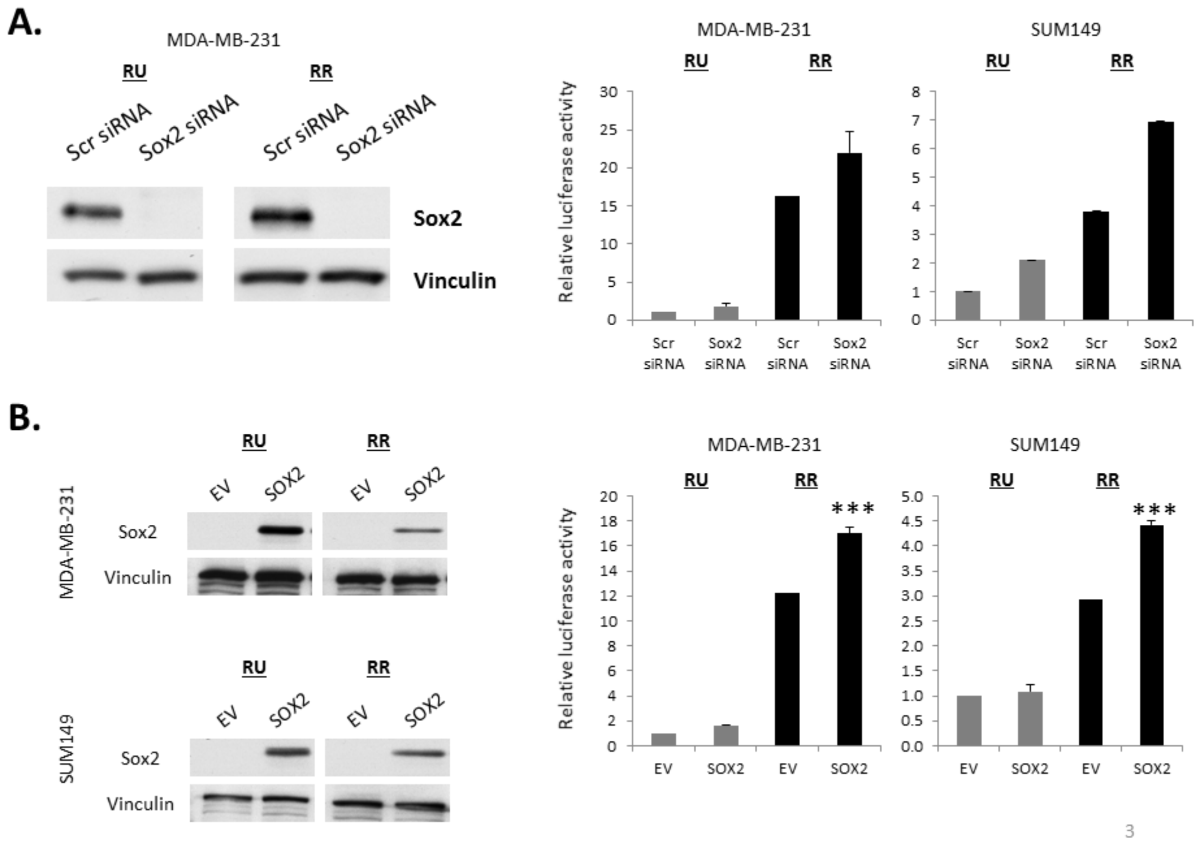

Supplemental Figure 3: Sox2 is not a major contributor in driving the SRR2 reporter. A. Relative SRR2 luciferase activity results following a 48-hour 20 nM Sox2 siRNA treatment of ER- RU and RR cell lines. Western blots demonstrating Sox2 knockdown efficiency in Sox2-expressing MDA-MB-231 cells are shown. **B.** Relative SRR2 luciferase activity and western blot results of ER- RU and RR cell lines transfected with 1.5 µg of empty vector (EV) or *SOX2*-expressing plasmid.
